# Ethical challenges in acute geriatric care and advance care planning: a nested qualitative study

**DOI:** 10.1007/s41999-026-01563-2

**Published:** 2026-07-28

**Authors:** Karin Berg Hermansen, Trygve Johannes Lereim Sævareid, Reidar Pedersen, Rigmor Einang Alnes, Linn Brøderud, Maria Romøren, May Helen Midtbust

**Affiliations:** 1 Department of Health Sciences, Aalesund Faculty of Medicine and Health Sciences, Norwegian University of Science and Technology, Box 1517, 6025 Aalesund, Norway; 2 Centre for Medical Ethics, University of Oslo, Box 1130, 0318 Blindern, Oslo Norway; 3 Department of Health and Nursing Science, University of Agder, Box 1130, 0318 Blindern, Oslo Norway; 4 Department of General Practice, Institute of Health and Society, University of Oslo, Box 1130, 0318 Blindern, Oslo Norway

**Keywords:** Ethics, Advance care planning, Older patients, Hospital, Health personnel, Qualitative

## Abstract

**Purpose:**

Ethical challenges are common in geriatrics, and moral dilemmas and conflicting interests can arise in advance care planning (ACP). Respecting the wishes of frail, acutely ill older patients while acting in their best interests can be challenging. This study was nested within a cluster randomized controlled trial aimed at implementing ACP in acute geriatric hospital units. The aim of this study was to explore clinicians’ experiences of ethical challenges in geriatric units before and during this implementation project.

**Methods:**

Eight semi-structured interviews—four focus groups and four dyadic interviews—were conducted with a total of twenty-four participants, including implementation teams and other clinicians from the six intervention units. These participants had engaged in ACP with frail, acutely ill older patients and, preferably, their next of kin. The data were analyzed using Braun and Clarke’s reflexive thematic analysis.

**Results:**

Four themes were developed: (1) navigating prognostic uncertainty while sustaining hope, (2) providing information that enables understanding and participation, (3) managing family disagreements and emotional strain, and (4) preserving dignity towards the end of life. The findings indicate that ethical challenges were often mitigated rather than intensified through ACP conversations.

**Conclusion:**

Ethical challenges in acute geriatric care arose when patient autonomy was limited by cognitive impairment, unclear preferences, or family disagreement. Earlier preparation of patients and next of kin through ACP could facilitate early identification of potential disagreements and provide a stronger foundation for dialogue in acute situations.

**Trial registration:**

ClinicalTrials.gov Identifier: NCT05681585. Registered 03.01.23.

**Supplementary Information:**

The online version contains supplementary material available at https://doi.org/10.1007/s41999-026-01563-2.

## Key summary points

**Aim:**

To explore clinicians’ experiences of ethical challenges in acute geriatric units before and during the implementation of advance care planning conversations.

**Findings:**

Four central challenges were developed: navigating prognostic uncertainty, enabling understanding and participation, managing family disagreements, and preserving end-of-life dignity. These challenges were mitigated rather than intensified through advance care planning conversations.

**Message:**

Integrating structured advance care planning into routine geriatric practice may help reduce ethical challenges and support clinical decision-making in acute care settings.

**Supplementary Information:**

The online version contains supplementary material available at https://doi.org/10.1007/s41999-026-01563-2.

## Introduction

Acutely ill older adults and their next of kin are frequently underinvolved in decisions regarding treatment and care [1]. Multimorbidity increases with age and is associated with reduced quality of life [2] and higher rates of hospitalization [3]. This study draws on Beauchamp and Childress’s four principles of biomedical ethics, respect for autonomy, beneficence, non-maleficence, and justice, as a framework for conceptualizing ethical challenges [4]. Ethical challenges arise when there is doubt, uncertainty, or disagreement about the right course of action and may also be described as conflicts between values [5]. In geriatrics, systematic reviews indicate that ethical challenges often arise in relation to the involvement of next of kin, choices concerning life-sustaining treatments, and the risks of both overtreatment and undertreatment [6]. Impaired decision-making capacities may lead to further ethical challenges [7].

Advance care planning (ACP) enhances respect for patient autonomy and shared decision-making [8, 9] and is widely regarded as an integral part of the continuum of care planning [10]. The primary goal of ACP is to support patient reflection and communication regarding personal values and preferences for future health care [11]. ACP has been associated with end-of-life communication [12]; reduced caregiver burden; improved alignment among clinicians, patients, and next of kin; and reduced clinician distress [13]. However, ACP conversations with frail, seriously ill older patients in hospitals may also give rise to ethical challenges for the clinicians who initiate and conduct them. Ensuring that older patients understand their medical condition and the implications of treatment options is essential yet may be complicated by cognitive impairments or communication barriers [14]. A review further showed that patient or next of kin preferences may conflict with the medical team’s assessments of the patient’s best interests [15].

ACP operationalizes ethical principles by creating a structured process for informed, value-based decision-making and balancing ethical considerations in future care scenarios. For example, ACP may safeguard patient autonomy when individuals are no longer able to actively participate in decisions regarding their care [16, 17]. ACP is relatively new in Norway, but national documents highlight it as an important tool for enhancing user participation [18, 19]. In Norway, advance directives are not legally binding. However, when patients lack decision-making capacity, previously expressed wishes, including preferences to limit life-prolonging treatment, should be respected if known to next of kin or clinicians [19]. At the same time, clinicians retain responsibility for professional decision-making [20] and are not obliged to provide medically futile treatment.

The present study was a qualitative interview study of clinicians, nested within a cluster randomized controlled trial aimed at implementing ACP in the routine care of acutely admitted patients in geriatric hospital units [21]. These patients often have a limited prognosis, significant prognostic uncertainty, and variable capability to engage in discussions about preferences and values. Despite its potential to align treatment decisions with patient values and preferences, ACP remains underutilized among frail older adults in hospital settings [22]. Although clinicians report positive attitudes toward shared decision-making, its adoption in practice remains low [23, 24]. A pre-implementation survey also revealed limited incorporation of patient preferences into healthcare decision-making [25].

Although the ethical challenges in geriatrics have been described in the literature, little is known about how clinicians navigate ethical tensions when implementing ACP in acute geriatric units. We anticipated that implementing ACP would involve ethical challenges, and identifying these challenges was essential for understanding how they influenced ACP practices and for developing strategies to overcome them. This study contributes novel knowledge by identifying, analyzing, and comparing such challenges before and during the real-world implementation of ACP in hospital-based geriatric care. The following research questions guided the study: ‘What ethical challenges do clinicians experience in acute geriatric hospital units before and during the implementation of ACP conversations?’.

## Methods

Reporting was guided by the ‘Reflexive Thematic Analysis Reporting Guidelines’ [26].

### Study design and context

This qualitative study was nested within a cluster randomized controlled trial evaluating the implementation of ACP in the routine care of acutely admitted patients in geriatric units [21]. Twelve acute geriatric hospital units in the Southeastern Norway Health Authority were randomized to either the intervention (receiving implementation support) or control (care as usual) arm. The project included two complex large-scale interventions in ordinary services: a clinical intervention (ACP) and an implementation intervention. Full details of the interventions and methods have been previously published [21, 27]. Each of the six intervention units established an implementation team comprising a local coordinator (nurse or physician), a unit leader, nurses, and physicians. These resource personnel received initial education and training before being expanded to all clinicians in the participating units. The clinical intervention involved offering ACP conversations to frail older adults admitted to acute geriatric units, preferably together with their next of kin. Participation was voluntary and followed a two-step model in which patient preferences for information and involvement were assessed before addressing more detailed ACP topics [28]. Communication techniques that emphasized active listening, rather than providing information alone, were deliberately used. The physicians led the conversations while the nurses played a supportive role. Geriatric units specialize in caring for older patients with frailty and multimorbidity. They use a multidisciplinary approach to manage acute illnesses while addressing the functional status, guiding treatment decisions, providing symptom relief, and considering ethical issues.

### Participants and recruitment

All participants were employed as clinicians in their respective units. The coordinators from the implementation teams assisted in coordinating the interviews. Data were collected through interviews with both the implementation teams and other clinicians in the six intervention units. We aimed to include nurses and physicians with experience with ACP conversations. Participant characteristics are detailed in Table 1.

**Table 1:** Baseline characteristics of participants (*n* = 24)

	*n*	%
Age range		
25–34 35–44 45–54 55–64	7 10 4 3	29.2 41.6 16.7 12.5
Sex Female Male	18 6	75 25
Occupation Junior doctor Specialist in internal medicine Specialist in internal medicine + geriatrics Others Nurse	4 3 7 1 9	16.7 12.5 29.2 4.1 37.5
Years of clinical experience 1–3 years 4–9 years 10–15 years > 15 years	4 6 6 8	16.7 25 25 33.3
Years in attending unit < 1 year 1–3 years 4–9 years 10–15 years > 15 years	3 6 13 1 1	12.5 25 54.2 4.1 4.1
Role Implementation team Other clinicians	12 12	50 50

### Data collection

We developed a semi-structured interview guide (Additional File 1) based on prior research by the Centre for Medical Ethics [29], focusing on two main questions: (i) Can you describe situations in which you have encountered ethical challenges related to ACP within the unit? and (ii) What ethical challenges have you experienced in your day-to-day clinical work? The interviews were conducted between December 2023 and October 2024. Four researchers from the implementation study’s project group co-facilitated the interviews in pairs. KBH (ICU nurse, PhD Fellow) participated in six interviews, LB (RN, PhD Fellow) in six interviews, and TJLS (RN, Assoc. Prof.) and MR (MD and Assoc. Prof.) in two interviews each. Focus groups enabled the interactive exploration of participants’ perceptions and experiences [30], whereas dyadic interviews, used when participant numbers were limited, provided deeper insights than individual interviews [31]. In total, 24 participants participated in 4 focus groups (3–5 participants) and 4 dyadic interviews. The participants had attended between one and ten ACP conversations, with an average of four conversations. Interviews were conducted in the hospital meeting rooms within each unit and lasted an average of 85 min. The implementation process was also explored during these sessions. The interviews were audio-recorded and transferred to the University of Oslo’s Service for Sensitive Data (TSD) for verbatim transcription. Additionally, during the implementation period, the experiences of the implementation teams in the intervention units were explored through regular dialogue and network meetings.

### Analysis

The analysis was guided by Braun and Clarke’s reflexive thematic analysis approach [32]. The field notes written after each interview enriched the analysis by capturing contextual reflections and emotional nuances. The first author, KBH, engaged deeply with the data by listening to all audio recordings and reading the transcripts multiple times. Each interview was coded line-by-line using an inductive, data-driven approach, with an emphasis on identifying explicit meanings. The coding process was conducted under the supervision of MHM. The NVivo 14.0 software was used to facilitate coding and data organization. The codes were then grouped into preliminary themes, highlighting the recurring patterns of meaning. The research questions guided the development of themes. The preliminary themes were discussed collaboratively with REA, TJLS, and MHM before being shared with all authors. The thematic structure was refined through an iterative process of moving between the data, codes, and emerging themes. Written notes on the ethical challenges from the network meetings were included in the analysis. All authors contributed to the interpretation and finalization of the themes through ongoing analytical discussions.

### Reflexivity

The researchers involved in the implementation trial, KBH, TJLS, RP, LB, and MR, contributed to the implementation process. Although our clinical background and familiarity with the project provided valuable contextual insights, we acknowledge our embedded and non-neutral roles. To mitigate potential bias, we actively asked for critical opinions from the participants during the interviews to present the clinicians’ experiences and perspectives.

### Ethical considerations

This study was conducted in accordance with the principles of the Declaration of Helsinki [33]. The Norwegian Agency for Shared Services in Education and Research (SIKT) (805,491) approved this study. The Regional Committee for Medical and Healthcare Research Ethics (REC) reviewed the research project and granted an exemption for health services research (52,825). Written informed consent was obtained from all clinicians before the interviews.

## Results

Clinicians in acute geriatric hospital units experienced ethical challenges as an inherent part of their practice, often reflecting tensions between clinical judgment and patient autonomy. These challenges appeared to be mitigated, rather than intensified, through ACP conversations. We developed four themes in our analysis: (1) navigating prognostic uncertainty while sustaining hope, (2) providing information that enables understanding and participation, (3) managing family disagreements and emotional strain, and (4) preserving dignity toward end-of-life care.

### Navigating prognostic uncertainty while sustaining hope

Clinicians consistently described the prognostication of frail, severely ill older patients as complex and clinically uncertain. Unlike patients with conditions that have more predictable trajectories, such as cancer, these patients often live with multiple chronic illnesses and severe frailty, making outcomes difficult to anticipate:

*It is difficult. It is not easy to predict—not at all. Because we are rarely talking about a cancer diagnosis where a new type of chemotherapy might be tried. Very few of our patients are in that situation. We’re usually dealing with multiple chronic conditions and severe frailty in many cases. And what will be the next thing to affect them? That’s really hard to know*. *Physician ID 6.5*

Clinicians experienced that uncertainty generated tension between providing honest communication about expected disease progression and their desire to maintain a sense of hope. They reflected on the challenge of addressing functional decline without undermining the patients’ sense of possibility.

*Prognoses and unrealistic expectations, that can be difficult to manage. How do you address it without taking away people’s hope, while also helping them understand… what’s really going on. It might be important that they do understand and reflect on it… You don’t recover from heart failure or COPD at that stage. Physician ID 3.1*

Some clinicians reported that patients sometimes believed that recovery was possible, even in advanced stages of illness. Despite these difficulties, most emphasized the importance of offering realistic and honest communication with both patients and their next of kin. To navigate this sensitive terrain, some described using ‘roundabout formulations’—language that conveys seriousness without sounding hopeless. Many physicians emphasized the importance of structured phrasing strategies in ACP, considering them both positive and helpful and highlighting the value of practicing and refining language for sensitive conversations.

They emphasized framing the prognosis as uncertain yet communicating it in realistic terms. Clinicians acknowledged not only the moral responsibility embedded in these decisions but also the deliberate relational strategies they employed to sustain trust. These strategies were seen as essential for navigating uncertainty and preparing patients and their next of kin for what lay ahead.

### Providing information that enables understanding and participation

Clinicians considered prognostication alone inadequate to support patients in making well-informed decisions. They highlighted the need to provide information and guidance that supported patients in understanding treatment plans, risks, benefits, and care options in alignment with their individual understanding and preferences. Clinicians encountered challenges in balancing participation and safeguarding well-being.

*You have to adapt [the information]; it's important to bring out the patient's thoughts and perspective. But you also need to gently correct things if they’re completely off… It’s about using a mix of compassion and clinical judgment, hand in hand. Physician ID 3.2*

Clinicians emphasized the value of inviting patients to clarify their preferences during ACP conversations and noted that it was new to ask, ‘How much do you know, and how much do you want to know about your situation?’ Several clinicians emphasized the value of enabling expression, even when wishes may later need refinement.

*I think it’s much better that they get to say what they want. And then you can work a little with it… I think it’s solved if you just have time to talk about it. Physician ID 1.1*

Clinicians felt that they had to tailor information to the situation. It was often challenging to honor patients’ wishes when their preferred options, such as further chemotherapy, were considered unlikely to be beneficial. Some described strategies for aligning expectations with medical realities, building on accurate perceptions, and gradually correcting misconceptions without imposing decisions.

*But to fish for what they [the patients] know and build on what is right and what is wrong in the perceptions they have — that might be the best way to provide a reality check. Physician ID 3.1*

This approach met patients where they were, honored their values, and paced information so that it could be absorbed and acted upon. In one case, although the patient initially expressed a desire to pursue further treatment, the physician, supported by the patient’s next of kin during the ACP conversation, helped the patient understand that additional intervention would provide no clinical benefit.

Timing also emerged as challenging. Clinicians experienced that many patients appeared ready to engage in ACP, particularly shortly before discharge. However, fluctuations in cognition and health owing to dementia, infections, or medication effects sometimes made it difficult to judge whether patients could retain, recall, and weigh information sufficiently to make informed choices. Clinicians reflected on the tension between initiating conversations to respect the patient’s voice and postponing until capacity improved, being aware that opportunities could be lost if conversations were delayed.

### Managing family disagreements and emotional strain

Many clinicians had experience with patients who were often concerned about placing a burden on others, particularly their next of kin.

*I’m a bit worried that some patients hold back when their next of kin are present [in hospital]—that they don’t dare to say how things really are, that they’re tired… I suspect they downplay their condition, so their loved ones won’t get upset. Physician ID 3.2*

Clinicians encountered cases in which patients shifted their wishes to align with perceived family expectations rather than their own true preferences. In general, many clinicians encountered situations in which family members held differing opinions on the care of frail older patients.

*A bit more honest communication [with patients alone], direct and without worrying too much about others. There are times when it’s actually next of kin who push for overtreatment, and you can sense that in the patient’s vague responses. You can tell there’s some pressure influencing their answers, one way or the other. Physician ID 7.1*

These disagreements often led to conflicts and made the decision-making process more complex. Clinicians described facing ethical challenges when navigating between honoring patients’ expressed wishes and responding to family members’ concerns. In one instance, the patient expressed a desire to avoid aggressive treatment. However, the next of kin insisted on pursuing all possible medical interventions.

*It’s often next of kin more than the patients themselves, who push strongly for additional treatment. I’ve cared for many patients— 95, 96 years old—who say they feel done with life. And then their son, daughter, or grandchildren insist: 'Give them fluids, give them treatment'. Nurse ID 8.1*

This placed clinicians in a challenging position, requiring them to mediate between upholding the patient’s autonomy and addressing the family’s emotional and moral concerns. Some experienced that it often required extensive conversations, often with next of kin, particularly when patients were unable to speak for themselves. They acknowledged that, in practice, treatment sometimes continued beyond what they considered appropriate, as the next of kin expressed a desire for continued intervention.

*It’s not uncommon to face the ethical dilemma of continuing treatment longer than what one truly believes is appropriate. Physician ID 1.1*

Several clinicians experienced, in the unit in general, that the next of kin might ‘take over a bit’, set the agenda, and arrive with strong views about anticipated care needs. Clinicians suggested that such advocacy often arose from limited information regarding burdens and outcomes.

*Next of kin often want more than what we believe is appropriate. But that’s often because they’re not well informed and don’t fully understand the burden or what the treatment actually entails. Physician ID 2.1*

Bringing patients, next of kin, and clinicians together in an ACP conversation was regarded as more than a practical step; it created a space for dialogue where differences could surface, perspectives could be exchanged, and decisions could be shaped collaboratively. One nurse reported: *It is an advantage that we gather the next of kin in the ACP conversation with the patient* (ID 5.2).

*Next of kin seemed very satisfied after the conversation; it increased awareness for both the patient and the family and encouraged reflection on the fact that patient’s life is nearing its end. Nurse ID 8.2*

Physicians emphasized their responsibility to justify decisions grounded in a frailty-informed assessment and manage unrealistic expectations by explaining likely outcomes and potential harms.

*Ultimately, it’s we [the physicians] who make the decision—and we have to be able to justify it. That’s part of our role in the geriatric unit, assessing frailty and the overall clinical picture, which allows us to explain our reasoning in a way that’s easier for others to understand…*

*Or what kind of miracles the treatment might bring—or, in fact, what serious consequences could occur if you went through with it. Physician ID 3.1*

Clinicians linked conflict with caregiver fatigue, noting that moments of family distress overshadowed patients’ needs.

*Sometimes, the next of kin can be a bit harsh toward their mother simply because they’re exhausted. They’ve reached a point of emotional fatigue where they just can’t cope anymore. In those moments, the atmosphere can become tense and painful—the focus shifts almost entirely to the next of kins’ distress and exhaustion, overshadowing the patient’s needs. Physician ID 3.2*

Clinicians frequently faced the challenge of balancing patients’ preferences to remain at home with the practical and emotional burdens placed on the next of kin, while also assessing when institutional care might become necessary. This led to difficulties in conversations and mediation.

#### Challenging discharges with exhausted family caregivers

Clinicians experienced situations in which, despite agreement on the need for additional support, external resources were insufficient. They observed that the next of kin experienced substantial fatigue and emotional strain because of prolonged caregiving responsibilities.

*It’s simply not medically justifiable for a 90-year-old spouse—who is exhausted and unwell themselves—to function as a caregiver. No one should have to spend the final stage of their life* trapped in a caregiving nightmare. Nurse ID 4.2

Despite clinical assessments indicating that patients required placement in a nursing home because of escalating care needs, such a placement was sometimes not made available. A lack of access to appropriate long-term care services could place both the patient and their next of kin in a vulnerable position, with family members struggling to provide adequate support at home. The clinicians expressed concerns about the ethical and systemic challenges posed by what they perceived as delayed access to appropriate long-term care.

*We are faced with the lack of resources available to them once they leave our care…. That’s something I find increasingly ethically challenging—the scarcity of healthcare resources and follow-up options in the community. I feel that some patients don’t get the follow-up they need… and that worries me. Physician ID 7.1*

Delayed institutional care compromised the quality of care, created moral distress among clinicians, and may contribute to caregiver burnout.

### Preserving dignity towards end of life

Clinicians described the complexity of assessing whether continued treatment was medically justified or potentially futile and how these decisions aligned with the goal of preserving dignity and comfort toward end of life. Many reflected on the difficulty of balancing the instinct to preserve life and avoid unnecessary suffering.

*We have been present at many cardiac arrests, and we’ve often asked ourselves: should we continue or not? Physician ID 4.1*

Some clinicians experienced that discharge summaries often indicated that hospital readmission was not recommended for certain patients. However, many of these patients were still readmitted, particularly from nursing homes, an observation echoed by others.

*I’ve experienced that quite a few times, and I find it undignified—when older, seriously ill patients are brought in from nursing homes where they weren’t allowed to die peacefully. Then they arrive here [hospital], and we end up poking and prodding them, and it doesn’t help. That ends up being their final experience. Physician ID 4.3*

*And not least, for their next of kin— it’s incredibly distressing for them. The patient becomes like a ping-pong ball between systems during their final week of life, instead of being surrounded by calm and dignity. Nurse ID 4.2*

Clinicians reflected on the ethical challenges they faced in balancing the desire to prolong life with the consideration of quality of life, deciding when to cease interventions that may be medically futile and focusing on palliative care instead. Determining the appropriate level of treatment was essential but deeply complex, particularly when patient wishes were unclear.

*Where should we position ourselves here? We sometimes have an overfilled ICU and intermediate care unit, and an older patient who is very ill. Where should we… what level of treatment should we aim for? If we had an ACP conversation beforehand, it would have helped us a lot.* Physician ID 7.2

Several clinicians noted that prior ACP conversations could significantly support decision-making and reduce uncertainty. However, they noted that such decisions were not considered absolute and had to be reassessed regularly considering the patient’s evolving clinical situation. Some clinicians emphasized the importance of distinguishing between treatment limitations initiated in the context of a progressive illness and those introduced following an acute condition. Treatment limitations were not perceived as fixed truths but as decisions that needed to be revisited, particularly after an acute episode, with the recognition that patients’ wishes could change over time. This theme reflects the ongoing challenge of balancing intensive treatment with maintaining dignity.

## Discussion

This study provides novel insights into the ethical challenges experienced by clinicians in acute geriatric hospital units before and during the implementation of ACP conversations. Caring for frail older patients requires the navigation of a range of ethically complex situations. Our findings highlight four main challenges: balancing uncertainty and hope, supporting patient understanding and involvement, managing family disagreements and emotional strain, and maintaining dignity towards the end of life. Contrary to our initial assumptions, the findings suggest that ACP, as implemented in the main project, may help mitigate, rather than intensify, several of the ethical challenges clinicians encounter when acutely admitting multimorbid and frail older adults.

Our findings indicate that clinicians find prognostic uncertainty near the end of life particularly challenging. Nevertheless, they emphasize the importance of eliciting patients’ preferences and offering meaningful choices while carefully preserving hope. Han et al. concluded that uncertainty in health care was a ubiquitous, multifaceted, and significant challenge [34], and Collins et al. highlighted that uncertainty could act as a barrier to decision-making and ACP implementation. However, ACP is inherently intertwined with uncertainty and cannot be meaningfully separated from it [35]. Providing honest information regarding prognosis is a crucial starting point for ACP conversations [36]. When afforded time and a safe space to explore hopes and concerns together with the next of kin, patients often move toward a greater prognostic understanding [37]. Consistent with earlier research, templates with predefined phrases were helpful in supporting documentation [38]. Our findings may encourage clinicians to recognize the value of attentive listening to patients’ wishes and hopes toward the end of life, even in the absence of prognostic certainty.

Our findings further indicate that clinicians strongly value patient autonomy; however, clinical assessments often limit their full realization. Respect for autonomy, grounded in informed consent, is central to ethical care [39], but frequently comes into tension with beneficence and non-maleficence when choices risk harm [40]. This tension underscores the complexity of decision-making in acute geriatric settings. Clarifying preferences is essential; however, respect for autonomy and professional judgment must be considered and balanced against each other [41]. Norwegian law affirms the right of individuals with decision-making capacity to accept or refuse treatment [39]. However, our findings reveal an ethical distinction between refusal and the misconception of entitlement to specific interventions. Structured approaches, such as ACP, may help navigate these competing principles.

Impaired decision-making capacities can lead clinicians to experience ethical uncertainty. Frail, acutely ill older patients may face cognitive challenges, making it difficult to express their wishes and values. This vulnerability underscores the importance of relational autonomy and open communication, as emphasized by Voumard et al. [42]. Ethical principles rarely provide clear hierarchies; as Beauchamp and Childress argued, none is ranked a priori [43]. When patients struggle to articulate preferences, reliance on next of kin or prior ACP conversations becomes necessary, indicating the need to consider relational and social contexts when respecting autonomy.

However, our findings reveal frequent tensions between respect for patient autonomy and family demands. Clinicians navigated these challenges under uncertainty, striving to honor patients’ voices while ensuring realistic informed decisions. Different expectations regarding care pose significant challenges in clinical practice. Discharges were often challenging owing to exhausted family caregivers and limited resources, and a recent review noted that such situations involving vulnerable patients require time and clinical–ethical expertise [44].

Importantly, the clinicians in our study emphasized that involving the next of kin in ACP conversations could help prevent ethical challenges. Ethical challenges often arise when patients or their next of kin disagree with or misunderstand the medical team’s plan, frequently because of poor communication [14]. These situations become particularly complex when patients and their family members hold conflicting expectations. Although disagreements sometimes occurred, typically owing to insufficient information, their participation offered emotional support and helped patients process complex decisions, especially when treatment preferences diverged from their clinical judgment. These dynamics highlight the relational nature of decision-making. ACP conversations provide opportunities to clarify values, anticipate disagreements, and align expectations before crises occur, when time is limited and emotions are high. Fien et al. found that proactive ACP fostered a shared understanding and trust among patients, families, and clinicians [45]. Similarly, Rosca et al. reported that ACP promoted patient autonomy, enabled families to provide meaningful support, and assisted clinicians in delivering care aligned with patient values [46].

By promoting transparency and collaboration, ACP may help mitigate ethical challenges and support decision-making that respects patient autonomy, while also considering the concerns of the next of kin. When the next of kin is well informed and prepared, decisional conflicts may be reduced [47]. Rigby et al. emphasized that the focus should extend beyond specific treatment preferences to encompass the kind of life that a patient considers acceptable or unacceptable. Patient values, goals, fears, and concerns may provide essential guidance for decision-making [48]. To mitigate these challenges, clinical decisions should prioritize patient autonomy by ensuring that interventions do not exceed the scope of patients’ stated preferences.

According to our findings, many physicians had experience making decisions about life-sustaining treatment for very frail, severely ill older patients admitted from nursing homes. These situations frequently occurred in the absence of documented prior wishes, with medical records being either vague or silent on the matter. Our findings, which are consistent with those of Bond et al. [49], indicate that this responsibility becomes particularly complex when treatment outcomes are uncertain and patient wishes cannot be determined. In end-of-life care, clinicians face the challenge of balancing next of kin expectations with clinical realities, particularly when weighing life-sustaining interventions against quality-of-life considerations. Consequently, as Cardona-Morell et al. reported, non-beneficial treatments at the end of life remain common in hospitals, with negative repercussions for patients, next of kin, and clinicians [50], often owing to inadequate communication [51, 52].

The absence of ACP conversations may further intensify ethical challenges in the care of frail, severely ill older patients and deprive clinicians of opportunities to explore patients’ values and preferences. This gap can result in decisions that conflict with professional ethical standards, thereby contributing to moral distress. External constraints that compel clinicians to act against their ethical standards can further heighten this strain [53], underscoring the inherent tension clinicians face when balancing competing ethical principles [54]. In our study, clinicians emphasized the importance of discussing prognosis and care preferences with patients; however, Brøderud et al. identified time constraints and administrative demands as significant barriers to such conversations [27]. An inability to engage in ethically appropriate communication can lead to frustration and emotional strain.

Ethical challenges may arise even under optimal conditions, underscoring the need for clear communication, sound ethical reasoning, and patient-centered approaches. ACP conversations may be a tool for strengthening patient involvement and supporting decision-making. Our findings highlight the importance of a dynamic and ongoing process for preparing patients and their next of kin for future decisions.

### Strength and limitations

This nested qualitative study enabled an in-depth exploration of the clinicians’ perspectives and provided a unique opportunity to capture their views on ethical challenges immediately before and after the initiation of ACP conversations. These findings may have exploratory relevance in other contexts and may inform future large-scale studies. However, the participants’ experiences may not reflect clinical practice outside the intervention units. In addition to receiving education and training, participants may have had a particular interest in ACP, which may have influenced their perspectives. Furthermore, the clinicians’ experiences were explored shortly after the initiation of ACP conversations; therefore, the long-term effects of ACP were not examined.

## Conclusion

This study identified key ethical challenges in acute geriatric units related to balancing competing values and principles, particularly when patient autonomy is affected by cognitive impairment, a lack of previously expressed wishes, or family disagreement.

The results highlight the need for improved preparation of patients and next of kin for future care and treatment decisions. Strengthening such preparedness may foster meaningful dialogue, support patient autonomy, and improve the alignment between care decisions and patient preferences. ACP appears to play an important role in facilitating this process, underscoring the relevance of integrating structured ACP conversations into routine clinical practice to help mitigate ethical complexities in acute care settings.

## Supplementary Information

Below is the link to the electronic supplementary material.

Supplementary file1 (DOCX 46 KB)

## Data Availability

The dataset will be de-identified or deleted before the study ends in accordance with ethics approval and consent. If it is possible to de-identify and subsequently anonymize the data, they will be made publicly available.

## Abbreviations

ACP: Advance care planning
REC: Regional Committee for Medical and Healthcare Research Ethics
SIKT: Norwegian Agency for Shared Services in Education and Research
TSD: University of Oslo’s Service for Sensitive Data
