# Supplementary material for: Ethical challenges in acute geriatric care and advance care planning: a nested qualitative study
Source: Eur Geriatr Med. 2026 Jul 28;17(5):2453–63. doi: 10.1007/s41999-026-01563-2 (PMC13754969; doi:10.1007/s41999-026-01563-2)
Supplement: Supplementary file 1 [file 41999_2026_1563_MOESM1_ESM.docx]

**Additional file 1** Overview of questions in the interview guide

From: Ethical challenges in acute geriatric care and advance care planning: a nested qualitative study.

Implementing advance care planning in acute geriatric units – a cluster randomized controlled trial

INTERVIEW-GUIDE

An ethical challenge can be defined as a situation where there is doubt, uncertainty, or disagreement about what is right and good to do. It can also be described as a conflict of values.

- **Can you describe situations in which you have encountered ethical challenges related to ACP within the unit?** *If not:*
- **What ethical challenges have you experienced in your day-to-day clinical work?**

(Help questions: Was there anything that was difficult to talk about? How did you experience it? How were they handled?) For example:

- Patient vs. next of kin vs. health personnel wishes/opinions?
- How much to inform and involve?
- Possibility of creating too high expectations that cannot be met?
- **Are there specific topics in ACP that are challenging to discuss with patients or their next of kin?**

For example, about serious illness, frailty, prognosis/what may happen in the future, or

the patient's thoughts or wishes for the final phase of life.

- **What significance do you think the uncertainty related to prognosis (which is often considerable in serious illness and frailty) has for discussing prognosis?**
  - With health personnel?
  - With the patient and next of kin?
  - What about documenting something about the patient's prognosis in the medical record and discharge summary?
- **What significance do you think "what is important for the patient" has in ACP and in clinical decisions for frail older adult?**
- What about the patient's spiritual and existential needs (e.g., questions related to suffering and death, faith/religion/beliefs)
